# Perceptions of artificial intelligence system's aptitude to judge morality and competence amidst the rise of Chatbots

**DOI:** 10.1186/s41235-024-00573-7

**Published:** 2024-07-18

**Authors:** Manuel Oliveira, Justus Brands, Judith Mashudi, Baptist Liefooghe, Ruud Hortensius

**Affiliations:** 1 Department of Psychology, Utrecht University, Utrecht, The Netherlands; 2 Department of Industrial Engineering and Innovation Sciences, Eindhoven University of Technology, Eindhoven, The Netherlands

**Keywords:** Artificial intelligence, Large language models, Chatbots, Impression formation, Social evaluation, Morality, Competence

## Abstract

This paper examines how humans judge the capabilities of artificial intelligence (AI) to evaluate human attributes, specifically focusing on two key dimensions of human social evaluation: morality and competence. Furthermore, it investigates the impact of exposure to advanced Large Language Models on these perceptions. In three studies (combined *N* = 200), we tested the hypothesis that people will find it less plausible that AI is capable of judging the morality conveyed by a behavior compared to judging its competence. Participants estimated the plausibility of AI origin for a set of written impressions of positive and negative behaviors related to morality and competence. Studies 1 and 3 supported our hypothesis that people would be more inclined to attribute AI origin to competence-related impressions compared to morality-related ones. In Study 2, we found this effect only for impressions of positive behaviors. Additional exploratory analyses clarified that the differentiation between the AI origin of competence and morality judgments persisted throughout the first half year after the public launch of popular AI chatbot (i.e., ChatGPT) and could not be explained by participants' general attitudes toward AI, or the actual source of the impressions (i.e., AI or human). These findings suggest an enduring belief that AI is less adept at assessing the morality compared to the competence of human behavior, even as AI capabilities continued to advance.

## Introduction

The impressions we hold about Artificial Intelligence (AI), either based in reality or fiction, shape our interaction with any AI system. One clear example of how AI is increasingly impressing humans occurred in 2022, when a Google engineer developing a Large Language Model (LLM) perceived it as a sentient entity upon interacting with it (Tiku, 2022). Regardless of whether LLMs can be sentient or not, one thing is clear: it is becoming increasingly hard to distinguish information generated by AI from information generated by humans. The capabilities of current LLM-powered chatbots to mimic human behavior have been sufficiently impressive to compel social scientists to start using them as a tool to investigate human behavior, for instance, by prompting the system to produce responses to simulated scenarios (Binz & Schulz, 2023; Dillion et al., 2023). While much is understood about how people attribute a mind to artificial systems (e.g., AI, robots or virtual humans) (Gray et al., 2007; Shank et al., 2019; Wegner & Gray, 2017), it remains less clear how state-of-the-art AI systems are perceived by humans in terms of their capabilities to *simulate human social cognition*. Do people believe that an AI is capable of judging behaviors on core dimensions of social perception? Here, we investigate whether people expect the capabilities for social cognition to be similar between AI and humans.

Current advances in AI technology make it increasingly difficult to distinguish between what is artificial and natural in our social environment. This is supported by studies showing a substantial decrease in the human ability to distinguish natural faces from artificial faces generated with state-of-the-art AI, such as the so-called *deep fakes* (Nightingale & Farid, 2022; Tucciarelli et al., 2022). In fact, AI-generated faces can sometimes be perceived as more real than actual natural faces (Tucciarelli et al., 2022). The same difficulty to discriminate between human and AI origin was observed for written text (Darda et al., 2023; Gunser et al., 2021), poetry (Hitsuwari et al., 2023; Köbis & Mossink, 2021), and artworks (Gangadharbatla, 2022). This inability to accurately discriminate between a human and an AI can be related to people’s preconceptions about AI capabilities, and how these steer attention towards stimulus properties that may or may not be diagnostic of its origin.

In line with this idea, studies have shown how flawed heuristics based on intuition interfere with people’s ability to accurately judge the origin of verbal self-descriptions (i.e., human or AI-generated) (Jakesch et al., 2023). In fact, recent explorations of the ability of LLMs to simulate human behavior demonstrated that these models can produce highly convincing humanlike responses in domains such as moral judgments (Dillion et al., 2023), political and economic decision making (Argyle et al., 2023; Horton, 2023), and several classical tasks designed to study human cognition (Binz & Schulz, 2023). Studies like these are likely to be the first of many using human capabilities as a benchmark to evaluate the humanlike capabilities of increasingly sophisticated AI systems across time.

The current work proposes to focus, not on examining how human ability may be objectively simulated by AI, but on how humans naively assess the capabilities of AI to assess human behavior. In other words, we are mainly interested in understanding the extent to which people expect AI to be as capable as humans of formulating judgments based on observed human behavior—regardless of whether such judgments are accurate or not (i.e., forming subjective impressions about character or personality). Given the infinitude of possible behaviors humans can perform, the task of systematically addressing perceptions of behaviors and how people make sense of them might seem challenging at first, as there is a wide range of categories where human behaviors can be classified into. A reasonable approach to overcome this initial challenge is to focus on the basic dimensions that summarize most of the variability in how people describe their impressions about each other—which are based on behavior and other cues such as appearance (Borkenau, 1986; Freeman & Ambady, 2011; Gilbert, 1998; Stolier et al., 2018). In this regard, decades of research in social cognition highlight two important dimensions of social judgment that describe the type of information that humans find most relevant when socially evaluating each other: morality and competence.^1^ In the context of social evaluation, the dimension of morality encapsulates information (i.e., traits, behaviors) that cues the target’s intentions (e.g. friend or foe?), whereas the dimension of competence captures information that cues the target’s ability to enact their intentions (Abele et al., 2008, 2021; Brambilla et al., 2011; Fiske, 2018; Goodwin et al., 2014; Rosenberg et al., 1968; Wojciszke, 2005). These dimensions are theorized to represent the underlying structure of people’s latent beliefs about how trait categories (e.g., honest, intelligent, dominant, untrustworthy) relate to each other, which in turn serve as the lenses through which the perceiver interprets incoming social information such as behaviors and appearance (e.g., Freeman & Ambady, 2011; Gilbert, 1998; Rosenberg et al., 1968). Despite being considered theoretically independent, each dimension is positively and linearly correlated with valence (Oliveira et al., 2020; Suitner & Maass, 2008) meaning, for instance, that a behavior judged to be high (or low) on morality is also perceived as more positive (or negative). This reflects how valence is inherently intertwined with these dimensions (Fazio, 2007; e.g., Kervyn et al., 2013; Osgood et al., 1957; Rosenberg & Olshan, 1970), itself an unsurprising aspect of social perception given our human proclivity to describe stimuli or events in terms of how good or how bad something is. The common association with valence is important to note, as there is a large body of research (e.g., Judd et al., 2005, 2019; Nisbett & Wilson, 1977; Suitner & Maass, 2008; Yzerbyt et al., 2008) documenting how perceptions of morality and competence of the same target person are often either positively or negatively correlated (i.e., halo or compensation effect, respectively) depending on the context of perception (e.g., Judd et al., 2019). This also applies to judgments of behaviors, as a given behavior can be and is often judged on both dimensions, and the valence attached to one of the dimensions may taint the valence of the other dimension either through an assimilative (i.e., halo effect) or contrasting (i.e., compensation) process (Kervyn et al., 2012; Yzerbyt et al., 2008). Thus, although we highlight and focus on morality and competence as the main dimensions to focus on in the present studies, we additionally take steps to control and monitor the role that valence of social information may play in the current studies.

Understanding the extent to which we ascribe human judgment abilities to AI can help us better understand the degree of acceptance of AI across different domains of human activity. For example, it could be argued that the extent to which we trust in AI’s capacity to act as a therapist should depend on a belief in its ability to understand how humans experience emotional states. Prior research on mind perception has shown that humans ascribe less ‘mind’ to artificial entities (e.g., robots and autonomous computer systems) than to other humans (Bigman & Gray, 2018; Gray et al., 2007). Such entities are perceived to possess a moderate ability for reasoning, planning, and enacting intentions, but less ability to experiencing states such as sensations (e.g., pain, taste, pleasure) emotions (e.g., anger, fear, surprise) or moods (e.g., anxiety, euphoria) (Gray et al., 2007; Wegner & Gray, 2017). Importantly, the perceived lack of ability to experience emotion appears to underlie a human aversion to moral decision-making by machines (Bigman & Gray, 2018). Such an aversion aligns well with prior work showing how emotional experience is a requirement for moral judgment (Cameron et al., 2015; Greene et al., 2001; Haidt et al., 1993). These insights from the literature lead us to the prediction that humans will presume AI to be ill-equipped to judge humans on morality.

In regard to the ability of AI to judge human competence, however, it could be argued that the assessment of the ability to carry out plans and intentions relies more often on observable information and objective criteria (e.g., ranking performance in a marathon using time and distance measurements, calculating number of correct responses in an exam) than on emotional experience. Lending support to this possibility, previous studies found that humans rely more on algorithmic advice for objective tasks than for tasks requiring emotional capabilities (Castelo et al., 2019; Dijkstra, 1999; Lee, 2018). Such findings may be partially explained by a perception of algorithms—including AIs—to be capable of effectively judging actions that can be more objectively analyzed without the need to factor in emotional experiences.

The present paper focuses on the question of whether there is a general tendency for people to believe that an AI is less capable than humans to judge human behavior on morality than on competence. In particular, we were interested in investigating how a person’s preexisting beliefs about AI and human capabilities might indirectly express themselves in a scenario where the person is faced with the task to estimate the extent that social information is of artificial or human origin. To do so, we designed different variants of an experimental task to examine the extent to which people estimate that judgments of morality were more likely generated by a human (vs. an AI), and that judgments of competence were more likely generated by an AI (vs. human). In this study, we used written pieces describing impressions formed about previously validated behaviors varying in the degree to which they relate to morality or competence (Mickelberg et al., 2022). While doing so, we additionally controlled for how positively and negatively these behaviors were seen in both dimensions, bearing in mind the natural entanglement between the perception of how a behavior is located along a dimension spectrum (e.g.,) and how it is evaluated in terms of valence (e.g. low morality is more negative and high morality is more positive) (Rosenberg & Olshan, 1970; Rosenberg et al., 1968).

In line with previous findings showing that people perceive AI as an entity with a low ability to experience suffering, and therefore to be ill-equipped to reason about morality (K. Gray et al., 2012), we expected that people would estimate moral judgments about human behavior as less likely to have been generated by an AI. In turn, we expected that people would estimate competence-related judgments of human behavior as equally likely to have been generated by humans or AI, assuming that both have comparable ability to judge performance on the basis of deduction from objective information. In this sense, competence-related judgments are assumed to require less emotion. For example, judging whether a person is competent at chess can be judged on the basis of how often they win against challenging opponents, but judging how immoral stealing a car is requires the ability to empathize with the harm done to the car’s owner and the transgressor’s state.

Beyond the focus on the perceptions of AI capabilities to mimic human social judgments, we highlight that the current studies capture data from unique times in the history of AI development, i.e., before and after widespread exposure to LLM-powered AI chatbots such as ChatGPT (OpenAI, 2022) and allow to explore the importance of exposure on perceptions of AI (Hortensius & Cross, 2018). By February 2023, ChatGPT was one of the fastest growing platforms in history (Hu, 2023), with monthly visits to the website (chat.openai.com) growing from an estimated total of 616 million in January 2023 to 1.8 billion in May 2023 (Ruby, 2023). Data collected during this period offered us the opportunity to explore the extent to which exposure to this technology has any influence on how people perceive the capabilities of AI for social judgments.

### Overview of studies

**Fig. 1:**
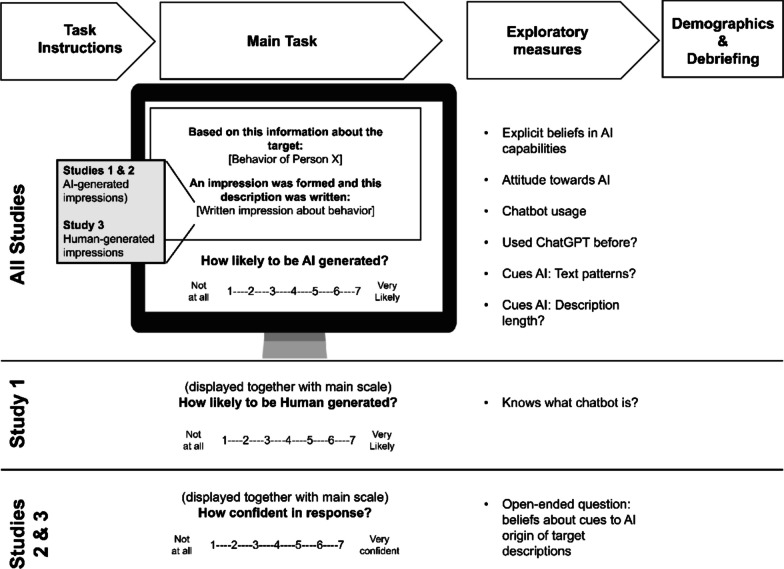
Overview of design and measures of all studies

We conducted three studies to systematically investigate people’s lay perceptions of AI’s capability to judge human behavior along two central dimensions of social judgment: competence and morality. In all studies, we employed an experimental task whereby participants read descriptions of impressions formed about specific human behaviors and indicated how likely they thought a given impression was written by AI (Fig. 1). Unbeknownst to the participants, the target items (i.e., written impressions based on a given behavior category), were all AI-generated (Studies 1 and 2) or human-generated (Study 3). Our primary goal was to test and find support for the following hypothesis:

H1: When participants are unsure if judgments about human behavior related to morality or competence were generated by an AI or a human, they will estimate judgments about competence as more likely to have been generated by AI. Conversely, participants will estimate that judgments of morality are less likely to have been generated by AI.

To gain a more nuanced understanding about domain-related perceptions of AI capabilities, we additionally explored a set of questions related to this hypothesis: (1) Is there any impact of the valence of a stimulus on estimations about its AI or human origin (e.g., judging AI’s capabilities to judge someone as moral vs. immoral)? (2) When explicitly asked about it, do people report believing that AI is more capable to judge the competence than the morality of human behavior?; and (3) Does the increased public awareness and exposure to advanced AI like ChatGPT have any impact people’s perceptions about AI capabilities to judge behavior on morality and competence? Beyond these questions, we additionally explored how the results could be related to variables such as: general attitude towards AI, confidence in one’s own estimations of artificial origin, reported usage of ChatGPT or chatbots, true origin of stimuli (i.e., AI or human), or individual opinions about cues to AI origin.

Study 1 was conducted shortly after the launch of ChatGPT (January 2023), and aimed to test our hypothesis (H1) that people would be more inclined to attribute AI origin to judgments of competence-related behavior compared to judgments of morality-related behavior. The results supported this hypothesis, by showing a statistically significant difference in the expected direction, i.e., participants rated competence-related judgments as more likely to be of AI origin compared to morality-related judgments (see Results). However, while exploring if the valence associated with the stimuli had any impact on the results, an unexpected interaction between the dimension and valence emerged, suggesting that the tendency to attribute AI origin to the stimuli was prominently occurring for judgments of positive behaviors. Within this study, we additionally verified the extent to which rating a stimulus as more likely to be of AI origin meant (to the participants) the same as rating the stimulus as less likely to be of human origin (i.e., that estimating AI origin and human origin represented two opposite poles of the same judgment dimension). Given the small sample size of Study 1 and its reliance on convenience sampling, it remained unclear to what extent the finding could be a false positive (Type I error). Thus, in Study 2 we aimed to replicate the results using a larger sample, sufficiently powered to detect both effects encountered in Study 1. Based on insights gained from Study 1, Study 2 introduced some updates to the set of exploratory measures of which we highlight a new open-ended question assessing personal beliefs about cues to AI origin. The results from Study 2 only replicated the (unpredicted) interaction between dimension and valence. In an attempt to understand if the true source of the stimulus materials could be driving the inconsistent results across studies, Study 3 aimed to replicate Study 2 with human-generated stimuli (i.e., impressions about the same behaviors written by independent human raters) instead of the AI-generated stimuli. Like Study 1, results from Study 3 supported our main hypothesis (H1), but the interaction between dimension and valence did not achieve significance, despite exhibiting a similar pattern to the previous two studies (i.e., effect was more prominent for positive information). In all studies the exploratory measures allowed us to understand what variables could be driving these effects.

Using the data from all studies, we explored how exposure to advanced AI affected the results. We assessed this in two ways: by exploring if the passage of time since ChatGPT was launched or if the amount of (self-reported) usage of chatbots like ChatGPT would impact the effects in any way. Although we made no specific predictions, we considered it reasonable to assume that the rising societal prominence of LLMs or the actual exposure to powerful chatbots would have some influence in perceptions of AI capabilities over time, by a process of belief updating through interaction with the technology.

## Method

### Open science statement

Data and materials can be found on this paper’s project page on the Open Science Framework: https://osf.io/r2t7b/.

### Time of data collection

Data for Study 1 was collected during January 2023 shortly after the first public release of Open AI’s ChatGPT on November 30, 2022. Data for Study 2 was collected 3 months later during April 2023 (after ChatGPT March 23 update was released). Data for Study 3 was collected shortly after Study 2, between May and June 2023 (after ChatGPT May 3 update was released).

### Participants

For Study 1, a convenience sampling method was used to recruit 20 adults (11 male, 8 female, 1 undisclosed gender; age range: 24–64 years, median = 28, IQR = 9.5, undisclosed age = 1; residing in The Netherlands = 17, Japan = 1, United States = 1, undisclosed = 1; all fluent in English) from the social network (i.e., friends, family, acquaintances) of one of the co-authors who was at the time an undergraduate student of AI. After completing the study, fourteen participants (70%) reported never having used Open AI’s ChatGPT prior to the study (vs. 30% who had used it). Half of the participants reported having used chatbots between 1 and 10 times before, eight participants reported having used chatbots more than 10 times, one participant used them at least one time, and another never used them before. For Study 2**,** 140 participants (70 male, 67 female, 1 non-binary / third gender, 2 undisclosed gender; age range: 19–70 years, median = 26, IQR = 8.25; residing in South Africa = 46, Poland = 24, and Portugal = 26, and 14 other countries = 44; fluent in English) were recruited via Prolific Academic (prolific.co) to participate in an online study. For Study 3**,** a convenience sampling method was used to recruit 40 participants (9 male, 30 female, 1 undisclosed gender; age range: 18–64 years, median = 24, IQR = 11.25, undisclosed age = 8; fluent in English and residing in The Netherlands = 28, Indonesia = 6, Sweden = 2, or undisclosed = 4) from the social network of another of the co-authors, also an undergraduate student of AI at the time. The median duration of participation in the studies were 21.06 min (Study 1), 18.68 min (Study 2), and 18.62 min (Study 3). For transparency, we clarify that Study 1 and 3 were conducted as part of undergraduate projects with limited financial and time resources. Study 2, which recruited a significantly larger online sample, received additional resources.^2^

### Design

All studies followed the same 2 (Dimension: morality, competence) X 2 (Valence: positive, negative)^3^ within-subjects design, with perceived plausibility of generation by AI as the main dependent measure. In Study 1, an additional dependent measure assessing the perceived plausibility of generation by a human was included to clarify whether the extent to which estimating a stimulus to be AI-generated implies an estimation that it was not generated by a human, or vice-versa—that is, whether this scale should be considered unidimensional, ranging from Human-generated to AI-generated. In Studies 1 and 2, all stimuli (i.e., impression descriptions based on behavior statements) were AI-generated, whereas stimuli in Study 3 were human-generated. See Fig. 1 for an overview of the design of all studies and how they differ between them.

### Power considerations

We report sensitivity power analyses for Studies 1 and 3 (i.e., estimation of the smallest detectable effect size given the available sample size), and a priori power analysis for Study 2 (i.e., estimation of minimum sample size required to detect a specified effect size). All analyses were conducted using the Shiny application PANGEA (Westfall et al., 2014; https://jakewestfall.shinyapps.io/pangea/) and focused on the statistical power to detect a within-subjects main effect of Dimension (all studies), and an interaction between Dimension and Valence (Study 2 and 3), while factoring in participants and stimuli (N = 24) as random effects. In all analyses alpha was set to 0.05. For Study 1, the sensitivity analysis indicated that a sample of 20 participants allows for the detection of a main effect of Dimension (predicted) as small as *d* = 0.62 with a power of 80.6%, and an interaction effect between Dimension and Valence (exploratory) as small as *d* = 0.42 with a power of 80.2%. For Study 2, we aimed to achieve sufficient power to conservatively detect an interaction effect as small as *d* = 0.30, to compensate for the likely inflated effect sizes encountered in Study 1 (main effect of Dimension was *d* = 0.60, 95% CI [0.05, 1.14] and the interaction was *d* = 0.59, 95% CI [0.08, 1.08]). The a priori analysis suggested 140 participants as the minimum required sample size to detect the interaction with 80.1% power. We further note that the derived sample size would allow to detect a slightly less conservative estimate of the main effect, specifically, *d* = 0.37 with 81.1% power. For Study 3, the sensitivity analysis clarified that its sample size of 40 allows to detect an interaction effect as small as *d* = 0.36, with 81.8% power, and a main effect as small as *d* = 0.43 with 81.1% power.

### Materials

#### AI-generated impressions

All the impression descriptions used in Study 1 and 2 were AI-generated. Materials were generated in two steps. In the first step, 24 behavior statements previously pre-tested on perceived morality and competence were extracted from the impression formation stimulus set developed and validated by Mickelberg et al. (2022). Our aim was to manually select behaviors that were rated as high (i.e., positive) or low (i.e., negative) as possible on one judgment dimension (e.g., morality) while being as neutral as possible on the other judgment dimension (e.g., competence). This resulted in a total of four sets of stimuli, each with six behavior statements, namely: Competence High (CH), Competence Low (CL), Morality High (MH), and Morality Low (ML). In a second step, we used Open AI’s ChatGPT (December 15 version; OpenAI, 2022) to generate text descriptions of an impression formed about each behavior statement. To standardize the generation process, we systematically prompted ChatGPT to “Describe an impression about a person X who [behavior statement]”. Importantly, we followed a zero-shot prompting approach, meaning that we did not train the chatbot with example data. Some statements, however, exceptionally required follow-up adjustments. For instance, some statements explicitly mentioned they were generated by an AI system (e.g., “It is not appropriate for me to provide an opinion about […]. As an AI, I do not have personal opinions or biases…”). In such cases, we removed the parts of the text revealing the true source of the description. Some of the initially generated descriptions also contained cues that we believed to be strongly suggestive of AI origin, such as repeating text patterns (e.g., “Overall,…” as a closing summarizing sentence or the same opening sentence listing three attributes to Person X). To minimize the influence of these cues we prompted ChatGPT to rewrite them using the following prompt: “Rewrite this text with different keywords, a different text structure and without a summarizing sentence at the end”. Examples of the AI-generated impressions and respective behaviors are shown in Table 1 (all stimuli are available in the OSF repository).

**Table 1:** Examples of descriptions of impressions generated by AI or humans

Dimension and valence	Behavior	AI impression	Human impression
Positive competence	Person X solved a crossword puzzle in the newspaper	Solving a crossword puzzle in the newspaper may suggest that Person X is intelligent, knowledgeable, and has strong problem-solving skills. They may enjoy mental challenges and have a love of learning, as crossword puzzles often require a wide range of general knowledge and vocabulary. Person X may also be patient, as completing a crossword puzzle can take time and require persistence. Additionally, they may be detail-oriented and have good memory skills, as solving a crossword puzzle often involves recalling specific pieces of information and paying attention to small details	Solving a crossword puzzle can be difficult and time consuming. Person X presumably has a broad general knowledge and enjoys problem solving
Negative competence	Person X failed their driver’s license test for the fourth time	The fact that the person has failed their driving license test for the fourth time may suggest that they are not currently skilled at driving, or that they are having difficulty acquiring the necessary knowledge and abilities to pass the test. It is possible that this could be due to a lack of preparation or study on their part, or it could be the result of anxiety or nervousness during the testing process	When a person fails their driver’s test four times, it might be advisable to take more driving lessons. It might also be the case that person X faces driving anxiety and therefore needs a special driving test
Positive morality	Person X shaved their head when they found out their partner had cancer and required radiation therapy	This act shows that Person X is willing to make a significant personal sacrifice in order to support their partner during a difficult time. It reveals that Person X is deeply compassionate and cares deeply about the well-being of their loved ones. It also shows that Person X is willing to take concrete steps to show their support and solidarity, even if it means going through something difficult or uncomfortable themselves. In this situation, Person X appears to be a loving and selfless individual who is willing to go above and beyond to support and care for those they love	Radiation therapy can cause hair loss on the part of the body that is being treated. Shaving one’s head can be seen as an act of solidarity with the cancer patient. It is an act of kindness and extra love towards their partner who is having a hard time
Negative morality	Person X bribed a landlord to be the first to get their apartment repainted	They demonstrate immoral and dishonest behavior by attempting to bribe a landlord for a favor. Offering something of value in exchange for something that may not be within the bribed person's power or authority is known as bribing. In this case, Person X uses their own resources to try and influence the landlord's decision, potentially at the expense of other tenants who may have also requested the same favor. This type of behavior is not only unfair, but it may also be illegal as it involves using personal resources to gain an advantage over others through corrupt means	Offering a bribe to your landlord is an intentional dishonest act. When this leads to the apartment being repainted first, person X is deceiving their other neighbors

#### Human-generated impressions

All the impression descriptions used in Study 3 were human-generated. These descriptions were based on the exact same behavioral statements previously used for the AI-generated materials. Two independent human volunteers were recruited from the network of one of the researchers to generate impressions from the behavior statements. Each human volunteer generated impressions about half of the behavior statements in each of the four categories of statements defined by dimension and pole of valence (i.e., positive or negative competence, positive or negative morality). Thus, the input of two different human judges was counterbalanced within each category of behavioral stimuli (e.g., each human judged half of all positive morality behaviors). One key difference between LLM and human judgments relates to the fact that, while a LLM generates its output based on the basis of a vast amount of information on which it was trained upon about a given topic (e.g., millions of opinions about the act of stealing), a human generates judgments based on their unique perspective (e.g., one opinion about stealing). The expression of more unique perspectives on morality and competence are expected to be a feature of human-generated materials (Table 3). The decision to ask a male and a female had the aim to minimize potential gender-related differences in moral or competence judgments (e.g., previous work identified gender differences in moral reasoning; Fumagalli et al., 2010). Examples of the human-generated impressions and respective behaviors are shown in Table 1 with all stimuli available in OSF repository. No information about the research question was explicitly given to the volunteers, so as to not influence their judgments. It should be noted that we trusted the volunteers to generate their own impressions (i.e., without AI assistance).

### Dependent measures

#### Plausibility judgments

Study 1 included two versions of a scale of perceived plausibility of generation (of a given written impression description). One version of the question asked the participant “How likely do you think that this description was generated by an Artificial Intelligence?”. This is the main dependent measure in this study. The other version was in every way identical but asked if the generator was “…a human?”. The response scale ranged from 1 (Not at all) to 7 (Very likely). To ascertain whether participants’ responses on the two plausibility scales of Study 1 were indicative that participants considered the ‘plausibility of AI origin’ and ‘plausibility of human origin’ to represent different framings of the same question—i.e., whether their response reflects a unidimensional decision space ranging from human-generated to AI-generated—we calculated the correlation between the two plausibility scales (i.e., AI-generated vs. human-generated) for each participant individually, converted these to Fisher z transforms, computed their average, and finally converted the result into the average correlation between the scales. The average of the individual correlations between the two plausibility scales was large and negative, mean *r* = − 0.83, *SE* = 0.13, *t*(18) = − 6.31, *p* < 0.001. This suggests that the two scales tap into a single dimension of plausibility of generation, such that the higher the response in the AI generation scale, the lower we can expect the response to be on the human generation scale. All the analyses reported in Results use the responses of the scale assessing the plausibility of AI generation, as our main focus is on how people judge the capabilities of AI. In light of the correlation between the human and AI origin scales, Studies 2 and 3 only included the scale of perceived plausibility of generation by AI. In the context of the current studies, a judgment of plausibility of AI origin is best understood as a representativeness heuristic (Tversky & Kahneman, 1974), i.e., a heuristic that people use to infer the probability that attributes of a given event (e.g., written impression of a given behavior) fit with one’s prior knowledge about a given category (e.g., AI or humans). We consider these judgments to be a proxy measurement of people’s lay theories about human and AI capabilities.

### Exploratory measures

A set of additional questions were included after the main task was completed. All studies shared most of these items with some exceptions and small adjustments. Unless otherwise specified, the measures were used in all three studies (Fig. 1).

In Studies 2 and 3, the degree of confidence in each estimation of AI origin was assessed with the question item “How confident do you feel about your response above?”, using a scale ranging from 1 (Not at all) to 7 (Very confident). This measurement was added after Study 1 to facilitate the interpretation of participants’ estimations when these fall on the midrange of the plausibility of AI origin scale. One item was included to assess the general attitude towards AI (“What is your attitude towards Artificial Intelligence in general?”) on a scale ranging from 1 (Extremely negative) to 7 (Extremely positive). Two items explicitly assessed the extent to which participants believed that an AI is capable of forming judgments about someone’s competence, or the morality of their behavior. These items were formulated as: “How much do you believe that an Artificial Intelligence system, in the present day, is able to form a judgment about the [abilities of someone / how moral someone’s behavior is]?”, and a 7-point response scale ranging from 1 (Not at all capable) to 7 (Very capable). Participants were also asked if they have ever used Open AI’s ChatGPT before on a Yes–No response scale. In Study 1, participants were asked if they knew what a chatbot was using a Yes–No response scale, and “how many times before [the participant] interacted with a chatbot powered by Artificial Intelligence” on a nominal scale with four response options (viz. Never, At least one time, 1–10 times, More than 10 times). In Studies 2 and 3, a different 7-point response scale was used for the same question, ranging from 1 (Never used chatbots before) to 7 (Almost every day). Two items exploring the potential cues participants could have used to inform their plausibility judgments: one assessed “How much do you think the length of each description was an indication that it was generated by an A.I. or by a person?”, and the other “How much do you think text patterns or word use in the description was an indication that it was generated by an A.I. or by a person?”. Both questions shared a 7-point response scale ranging from 1 (Not at all) to 7 (Very indicative). In Studies 2 and 3, an open-ended question asked for the participant’s opinion about the “aspects or cues in the descriptions that made [them] suspect that a description was written by an A.I.”.

### Procedure

All studies were administered online using Qualtrics software (https://www.qualtrics.com). After reading the informed consent and agreeing to participate in the study, participants were presented with a welcome message that provided background information about AI and its current capabilities. In addition, the instructions included a brief description stating that the motivation behind the study was to investigate “how people evaluate information reflecting judgments about other people, when this information is generated by different sources, namely: humans and artificial systems (such as Artificial Intelligence algorithms)”, and informed that some descriptions would be presented during the task that were either generated by a human or by an AI. Unbeknownst to the participants, the descriptions in Studies 1 and 2 were all AI-generated, whereas in Study 3 the descriptions were all human-generated. Participants were instructed that their task on each trial would be to indicate how plausible they believed that a given impression description based on a behavior was generated by an AI (all studies). Prior to the main task, participants completed a practice block to get acquainted with the task. The main task consisted of 24 trials presented in randomized order. In each trial screen, there were three elements: a behavior statement, a description of the impression formed based on that behavior, and the two response scales (Study 1: plausibility of generation by AI or human scales, with up-down positioning of the scales counterbalanced between-subjects; Studies 2 and 3: plausibility of generation by AI scale always on top, and response confidence scale below). Two pieces of text, one above the behavior description (“Based on this information about the target:”), and another above the impression description (“An impression was formed and this description was written:”) were presented in each trial with the aim to continuously remind the participant that each description was based on a behavioral judgment. Importantly, participants were never explicitly informed about the category membership (i.e., morality or competence) of the behavior or impression description. After completing the main task, participants responded to a set of post-experiment questions, and provided demographical data (age, gender, and current country of residence). Finally, participants were thanked and debriefed about the purpose of the study. In addition, they were asked to not share the details of their participation with other potential participants.

### Data analysis

All analyses were conducted in R v.4.3.1 (R Core Team, 2022). The main analyses of this study examining the effects of the dimension of impression description on the perceived plausibility that it was AI-generated were conducted using linear mixed-effects models as implemented in the packages ‘lme4’ (Bates et al., 2015) and ‘lmerTest’ (Kuznetsova et al., 2017). Estimated marginal means and simple effects were calculated with the package ‘emmeans’ (Lenth, 2022). We followed a linear mixed-effects models approach instead of using the traditional ANOVA as these models take into account the variability of the estimated effects across participants and stimuli, thereby allowing for more nuanced and generalizable results (for more details see DeBruine & Barr, 2021). The use mixed models is further suggested by the intraclass correlation coefficient (ICC) of the mixed model of interest testing the effect of Dimension on plausibility responses, which suggests a reasonable degree of influence of group-level clustering on the overall data variability (ICC = 0.33). When specifying our models, we took into consideration the recommendation by Barr et al. (2013) to specify a maximal model, while at the same time adjusting it to reflect as closely as possible the research question of interest. The main model we specified tested the effect of the judgment dimension on the perceived plausibility of AI origin. In this model, Dimension (morality vs. competence) was specified as a fixed effect (within-subjects). As random effects, intercepts and slopes of Dimension were allowed to vary by participant and by stimuli (i.e., impression descriptions):

$$\begin{aligned}&\text{Perceived Plausibility of AI origin Dimension}\\ &\quad +(1 + \text{Dimension }|\text{ Participant})+(1 + \text{Dimension }|\text{ Stimulus}).\end{aligned}$$

The exploratory analyses exploring the interaction between Dimension (morality vs. competence) and Valence (positive vs. negative) on perceptions of AI origin, simply added the main effect of Valence and an interaction term Dimension x Valence:

$$\begin{aligned}&\text{Perceived Plausibility of AI origin Dimension * Valence} + (1 + \text{Dimension * Valence }|\text{Participant})\\ &\quad+(1 + \text{Dimension * Valence }| \text{ Stimulus}).\end{aligned}$$

In Study 1, we first ran the main model (Dimension only) and then explored the interaction model (Dimension * Valence). In Study 2 we ran only the interaction model, looking first at the interaction to verify if it was replicated under more optimal circumstances of data collection, and subsequently inspecting the main effect of Dimension within that model. In Study 3, we again ran only the interaction model. To be clear, however, only the main effect of Dimension is directly related to our main hypothesis (H1). Other effects should be regarded as exploratory.

The models exploring the effects of increased exposure to information about AI capabilities, and of the true origin of stimuli (AI- or human-generated), added either of these variables as an additional fixed effect interacting with Dimension*Valence depending on the analysis under focus. The variable “Study number” reflects the chronological sequence of the studies related to an increasing degree of public awareness about AI capabilities between January and June of 2023. These models were specified as:

$$\begin{aligned}&\text{Perceived Plausibility of AI origin [Study Number or Stimulus Origin] }*\text{ Dimension }\\ &\quad+ (1 + \text{Dimension }|\text{ Participant})+(1 + \text{ Dimension }| \text{ Stimulus}).\end{aligned}$$

The same rationale was followed for models with covariates:

$$\begin{aligned}&\text{Perceived Plausibility of AI origin [Covariate]} + \text{Dimension} \\ &\quad + (1 + \text{Dimension} | \text{ Participant})+(1 + \text{ Dimension }|\text{ Stimulus}).\end{aligned}$$

For increased readability of the results, we report the results of the mixed-effects models converted into an ANOVA format. Statistical significance of fixed effects and their interactions was assessed with F-tests using Satterwhaite’s method for estimating the degrees of freedom of the denominator (Luke, 2017). Please note that the degrees of freedom estimated using Satterwhaite’s method may look counter-intuitive compared to the ones calculated in more traditional variants of ANOVA. P-values below 0.05 were considered statistically significant. A false discovery rate (FDR) correction (Benjamini & Hochberg, 1995) was applied to the p-values of multiple comparison tests following any significant interaction effects, or main effects of factors with more than two levels. Unless specified otherwise, statistical tests are two-sided.

## Results

### Reliability of plausibility of generation judgments

We calculated Cronbach’s α to assess the internal consistency of judgments of plausibility of AI (or Human) origin. The calculation was performed at the level of plausibility judgment using each participant’s responses to the 24 stimuli, i.e., collapsing across the dimension and valence categories of stimuli. In Study 1, the reliability of judgments on the scale of plausibility of AI origin (α = 0.61) and on the scale of plausibility of Human origin (α = 0.64) were both low, likely a consequence of the low sample size. However, the reliability of judgments on the scale of plausibility of AI origin was higher in Study 2 (α = 0.93) and Study 3 (α = 0.84), which may more accurately approximate to the true reliability given the larger sample sizes they were derived from.

### Plausibility of generation by AI

**Fig. 2:**
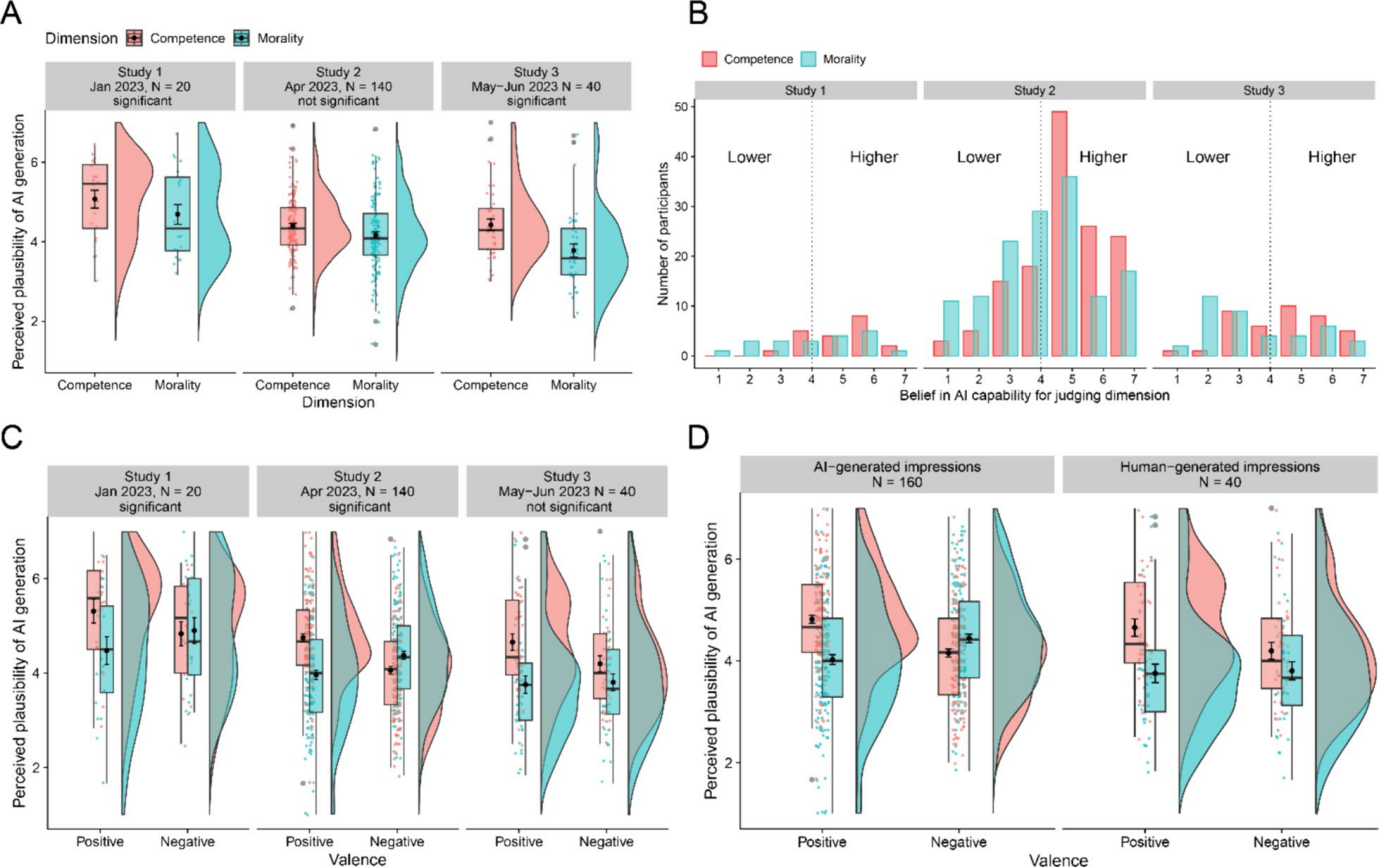
Overview of results from studies 1, 2 and 3 for perceived plausibility of AI origin in main task and self-reported beliefs in AI capabilities. *Note.* Any reference to statistical significance reflects whether an effect had a *p*-value < 0.05. Panel A: Plots of the main effect of the perceived plausibility of AI origin by Dimension of the behavior behind an impression description. Panel B: Explicit ratings of perceived capability of AI to judge morality and competence. Panel C: Interaction plots of the perceived plausibility of AI origin by Dimension and Valence of the behavior behind an impression description. Panel D: Interaction plots comparing the perceived plausibility of AI generation between AI-generated and human-generated stimuli by Dimension and Valence of the associated impression descriptions

Plots of main effects (Dimension) and interactions (Dimension x Valence) are available in Fig. 2A and C.

*Study 1.* In the first model including only Dimension as the fixed effect, the main effect of Dimension was significant, *F*(1, 15.168) = 5.47, p = 0.033, *ƞ*_*p*_^*2*^ = 0.265, 95% CI [0.000, 0.564], thereby offering support to our hypothesis (H1). Specifically, participants tended to attribute higher plausibility of AI origin to competence-related descriptions (*M* = 5.07, *SE* = 0.10) than to morality-related descriptions (*M* = 4.68, *SE* = 0.10). This main effect of Dimension remained significant when separate models included as a covariate either general attitude towards AI, *F*(1, 15.169) = 5.47, *p* = 0.033,* ƞ*_*p*_^*2*^ = 0.265, or self-reported exposure to ChatGPT, *F*(1, 15.168) = 5.47, *p* = 0.033,* ƞ*_*p*_^*2*^ = . 265. Subsequently, we explored the effect of valence in a second model including the interaction term between Dimension and Valence. The results revealed an unexpected significant interaction, *F*(1, 18.174) = 6.26, *p* = 0.022, *ƞ*_*p*_^*2*^ = 0.256, 95% CI [0.003, 0.537], indicating that whether a judged behavior was positive or negative on a dimension impacted the degree to which people were willing to believe that it was generated by an AI. Follow-up analyses revealed that participants found it more plausible that AI generated competence-related descriptions than morality-related impressions, but only when these descriptions were about positive behaviors on each dimension, i.e., positive competence (*M* = 5.31, *SE* = 0.15) and positive morality (*M* = 4.47, *SE* = 0.19), *p*_FDR_ = 0.020. Descriptions about negative behaviors on competence (*M* = 4.83, *SE* = 0.17) and morality (*M* = 4.90, *SE* = 0.15) were perceived as equally plausible to be AI-generated, *p*_FDR_ = 0.99. None of the other comparisons achieved significance (all *p*s > 0.05). The Dimension x Valence interaction remained significant when separate models included as a covariate either general attitude towards AI, *F*(1, 18.165) = 6.26, *p* = 0.022,* ƞ*_*p*_^*2*^ = 0.256, or self-reported exposure to ChatGPT, *F*(1, 18.177) = 6.26, *p* = 0.022,* ƞ*_*p*_^*2*^ = 0.256.

*Study 2.* In Study 2 we ran the model including the interaction term with the aim to verify if the interaction in Study 1 would again emerge (see Data Analysis). The main effect of Dimension was not significant, *F*(1, 20.400) = 1.78, *p* = 0.197, *ƞ*_*p*_^*2*^ = 0.080, 95% CI [0.000, 0.351], indicating that participants did not differentiate between morality- and competence-related descriptions in their responses. There was also no main effect of Valence, *F* < 0, *ƞ*_*p*_^*2*^ = 0.035, 95% CI [0.000, 0.297], indicating no differentiation between impressions about positive and negative behaviors. However, the significant interaction between Dimension and Valence was replicated, *F*(1, 17.772) = 10.70, *p* = 0.004, *ƞ*_*p*_^*2*^ = 0.376, 95% CI [0.053, 0.627], indicating that the valence of the dimension of the description impacted the belief in its AI origin. Follow-up analyses of the simple effects revealed two significant comparisons. First, replicating the findings in Study 1, participants estimated descriptions conveying positive judgments of competence (*M* = 4.75, *SE* = 0.06) as more likely to have been generated by AI compared to descriptions conveying positive judgments of morality (*M* = 3.96, *SE* = 0.08), *p*_FDR_ = 0.009. Second, positive competence descriptions were perceived as more plausible to be AI-generated than negative competence descriptions (*M* = 4.05, *SE* = 0.08), *p*_FDR_ = 0.018. None of the other comparisons achieved significance (all *p*s_FDR_ > 0.05). Additional exploratory analyses further clarified that the Dimension x Valence interaction remained significant after controlling for participants’ self-reported confidence in their plausibility responses, *F*(1, 18.12) = 11.08, *p* = 0.004. Similar to Study 2, the interaction remained significant when separate models were conducted including either general attitude towards AI, self-reported exposure to ChatGPT, or self-reported general chatbot exposure as a covariate (all these models shared the result for the interaction: *F*(1, 17.773) = 10.70, *p* = 0.004).

*Study 3.* Only the model with the interaction term was run for this study (see Data Analysis). The interaction between Dimension and Valence was not significant, *F*(1, 19.195) = 1.31, *p* = 0.267, *ƞ*_*p*_^*2*^ = 0.064, 95% CI [0.000, 0.337], indicating that the dimension and pole of the behavior associated with impressions did not significantly impact the plausibility judgments of AI origin. In comparison with the previous studies, we note that this interaction was nevertheless medium-sized, and its pattern was similar to the observed in Studies 1 and 2 (Fig. 2C). However, once again supporting our main hypothesis (H1), a large significant main effect of Dimension emerged, *F*(1, 24.33) = 7.30, *p* = 0.012, *ƞ*_*p*_^*2*^ = 0.23, 95% CI [0.012, 0.484], indicating that descriptions conveying judgments of competence (*M* = 4.42, SE = 0.12) were perceived as more likely to have been generated by AI compared to descriptions conveying judgments of morality (*M* = 3.78, SE = 0.11). No other effects were significant. Additional exploratory analyses further clarified that the main effect of Dimension remained significant after controlling for participants’ self-reported confidence in their plausibility responses, *F*(1, 23.91) = 7.09, *p* = 0.014. The main effect remained significant when separate models (with interaction term) were conducted including either general attitude towards AI, self-reported exposure to ChatGPT, or self-reported general chatbot exposure as a covariate (all these models shared the result for the main effect of Dimension: *F*(1, 24.33) = 7.30, *p* = 0.012).

### Exploratory analyses

#### Explicit measure of beliefs about AI capabilities

Across all three studies, participants consistently rated AI to be more capable of forming judgments about someone’s competence compared to judging how moral someone’s behavior is (see Table 2 and Fig. 2B). Additional one-way ANOVA analyses indicated no differences between the three studies regarding the average belief in AI capabilities for judging competence, *F*(2, 197) = 1.23, *p* = 0.295, *ƞ*_*p*_^*2*^ = 0.012, or judging morality, *F*(2, 197) = 1.80, *p* = 0.169, *ƞ*_*p*_^*2*^ = 0.018. Aggregating data across all three studies indicated that the explicit ratings of AI capabilities for judgments of morality positively correlated with the responses of perceived plausibility of AI origin for morality-related descriptions, albeit weakly so, *r* = 0.18, *t*(198) = 2.62, *p* = 0.009. This indicates that, to a small degree, the higher participants explicitly rated AI as capable for moral judgments, the more plausible they estimated AI to have been the generator of morality-related judgments during the main task (i.e., without having knowledge of the strong association of these descriptions to the morality dimension). In contrast, the explicit ratings of AI capabilities for judgments of competence were not significantly correlated with the responses of perceived plausibility of AI origin for competence-related descriptions, *r* = 0.10, *t*(198) = 1.48, *p* = 0.141. The explicit ratings of AI capabilities for judgments of competence and morality were, nevertheless, strongly positively correlated, *r* = 0.66, *t*(198) = 12.29, *p* < 0.001, indicating that, on average, the more people believed in the capabilities of AI to judge one dimension, the more they believed it to be capable of judging the other.

**Table 2:** Mean ratings of beliefs in AI capabilities for making morality versus competence judgments

Study	Capability for competence *M* (*SD*)	Capability for morality *M* (*SD*)	Cohen’s *d* [95% CI]	Paired t-test *p*
1	5.25 (1.12)	4.25 (1.71)	0.69 [0.05, 1.33]	0.007
2	4.97 (1.45)	4.22 (1.70)	0.49 [0.25, 0.73]	< 0.001
3	4.68 (1.53)	3.65 (1.79)	0.62 [0.17, 1.06]	< 0.001

#### AI-generated versus human-generated stimuli

Study 3 allowed us to explore whether the true origin of the impression descriptions (i.e., AI or humans) moderated the results. The analysis was conducted using combined data from all studies. We focused on the model with the interaction between Dimension and Valence given its emergence in two of the studies. An additional factor categorized the observations from Studies 1 and 2 (N = 160) as AI-generated, and the data from Study 3 (N = 40) as human-generated. The three-way Stimulus Origin x Dimension x Valence interaction was marginally significant, *F*(1, 198.003) = 4.34, *p* = 0.038, but small, *ƞ*_*p*_^*2*^ = 0.021, 95% CI [0.000, 0.076], indicating that the Dimension x Valence interaction differed between AI- and human-generated stimuli. The relevant follow-up analyses clarified that the difference between positive competence and positive morality was significant for both AI-generated, *p*_FDR_ < 0.001, and human-generated stimuli, *p*_FDR_ = 0.004. And that the difference between negative competence and negative morality remained non-significant for both AI-generated, *p*_FDR_ = 0.170, and human-generated stimuli, *p*_FDR_ = 0.151. Although this appears to indicate a similar pattern of the interaction found in Studies 1 and 2 is also emerging with human-generated materials (Fig. 2D), we call attention for the unbalanced sample sizes between the AI- and human-generated samples, and to the non-significance of the interaction effect when only data from Study 3 is analyzed. The three-way interaction appears then to be driven by other simple effects revealing a discrimination between poles of valence within the same dimension: When the stimuli were AI-generated, participants perceived positive competence stimuli (*M* = 4.77, *SE* = 0.06) as more likely generated by AI than negative competence stimuli (*M* = 4.10, *SE* = 0.07), *p*_FDR_ = 0.005, and positive morality stimuli (*M* = 3.97, *SE* = 0.07) as less likely generated by AI than negative morality stimuli (*M* = 4.39, *SE* = 0.06), *p*_FDR_ = 0.043, although marginally so.

#### Effects of time since ChatGPT launch and exposure to chatbots

**Fig. 3:**
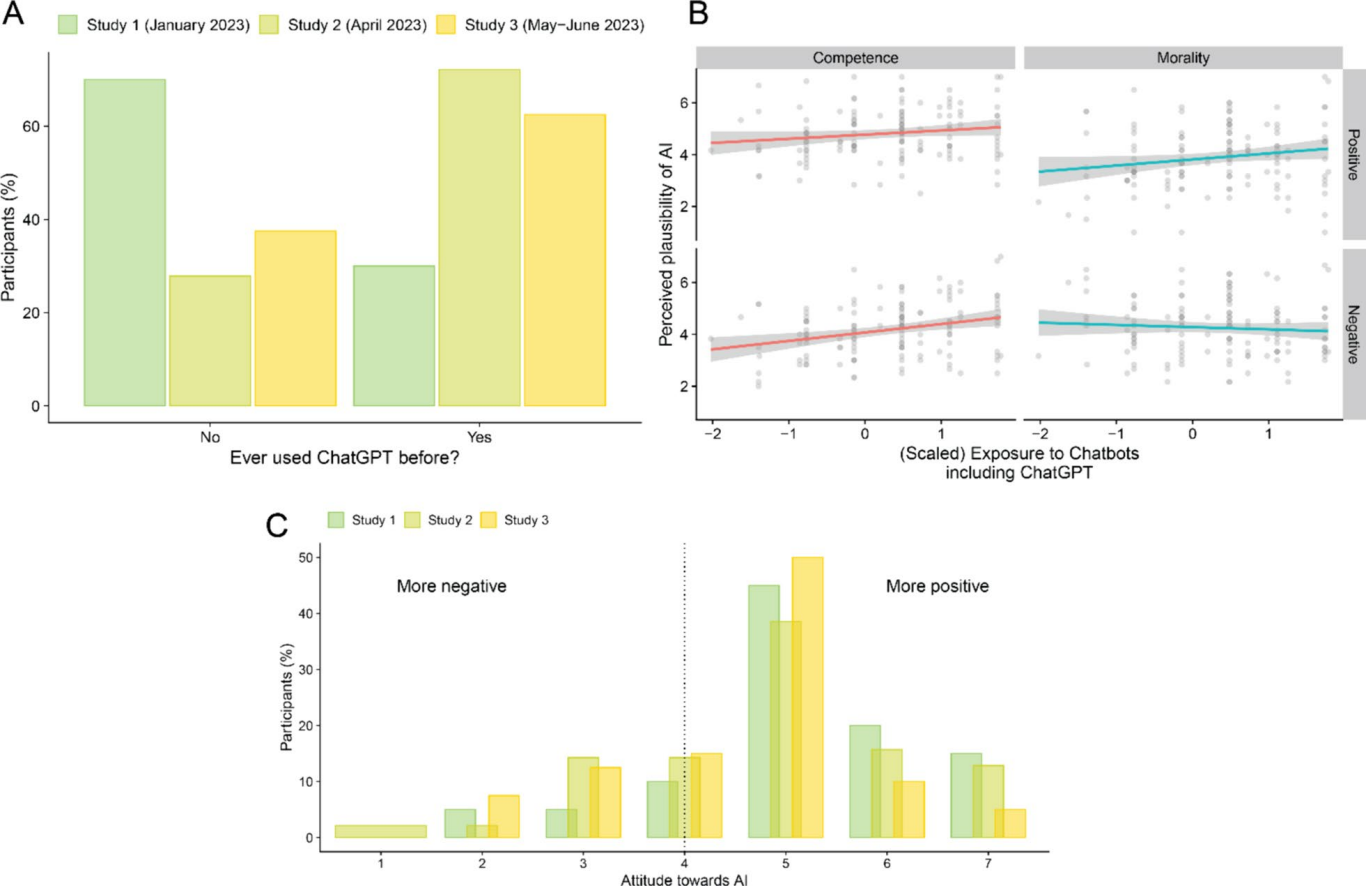
Overview of Results from Studies 1, 2, and 3 for Effects of Exposure to AI and General Attitudes Towards AI. *Note.* Panel A: Exposure of participants to ChatGPT across time and studies. Panel B: Relationship between self-reported exposure to chatbots including ChatGPT and responses of perceived plausibility of AI origin for descriptions of all Dimension by Valence categories (combined data from all studies). Panel C: General attitude towards AI

The self-reported awareness of LLM technology like ChatGPT substantially increased between Study 1 (January 2023; 30% exposure) and Study 2 (April 2023; 73% exposure), remaining relatively stable until Study 3 (May and June 2023; 62.5% exposure) (Fig. 3A). To investigate the impact of the increasing public awareness of LLM technology on our results, we aggregated the data of all studies and added study number as a fixed effect predictor to the model of perceived plausibility of AI origin. The sequence of study numbers directly reflects the time elapsed since the launch of ChatGPT, and likely increase in public awareness about the capabilities of LLMs. The Study Number x Dimension x Valence interaction was not significant, *F*(2, 196.999) = 2.31, *p* = 0.102, *ƞ*_*p*_^*2*^ = 0.023, 95% CI [0.000, 0.073], suggesting no evidence that the passage of time since the launch of ChatGPT had an impact on the interaction between Dimension and Valence. The Dimension by Valence interaction was significant, *F*(1, 23.38) = 8.33, *p* = 0.008, *ƞ*_*p*_^*2*^ = 0.263, replicating the difference in plausibility responses between positive competence (*M* = 4.78, *SE* = 0.06) and positive morality (*M* = 3.97, *SE* = 0.06), *p* < 0.001, and no difference between negative competence (*M* = 4.16, *SE* = 0.06) and negative morality (*M* = 4.31, *SE* = 0.06), *p* = 0.992. There was, however, a marginally significant Study by Dimension interaction, *F*(2, 197) = 3.13, *p* = 0.046, *ƞ*_*p*_^*2*^ = 0.031, indicating that the difference in mean perceived plausibility of AI origin between competence-related (*M* = 4.63, *SE* = 0.11) and morality-related (*M* = 3.99, *SE* = 0.11) descriptions was only significant in Study 3, *p*_FDR_ < 0.001, but not in Studies 1 (*p*_FDR_ = 0.112) or Study 2 (*p*_FDR_ = 0.113) where differences between dimensions depended on the valence of judged behaviors. Finally, there was a significant main effect of Study, *F*(2, 196.998) = 7.12, *p* = 0.001, *ƞ*_*p*_^*2*^ = 0.067, indicating that the overall mean perceived plausibility of AI origin in Study 1 (*M* = 4.88, *SE* = 0.23) was higher than in Study 2 (*M* = 4.29, *SE* = 0.06), *p*_FDR_ = 0.002, and Study 3, (*M* = 4.10, *SE* = 0.13), *p*_FDR_ < 0.001, while there was no significant difference between Studies 2 and 3, *p*_FDR_ = 0.179. We further explored how the self-reported degree of previous use of chatbots including ChatGPT (but not exclusively) related to responses of perceived plausibility of AI origin in our main task. First, we scaled and centered the chatbot exposure responses from all studies, to allow for the combination of the Likert type scale used in Study 1 with the corresponding bipolar scale used in Studies 2 and 2. Importantly, we included only the participants who reported having used ChatGPT prior to their participation (*n* = 132 out of 200). We found a positive correlation between (scaled) chatbot exposure and the tendency to perceive descriptions as more likely to be AI-generated, *r(*130) = 0.19, *p* = 0.028. Next, we ran a model similar to the one reported above, by replacing the study number predictor with (scaled) chatbot exposure. The Chatbot Exposure x Dimension x Valence interaction was significant, *F*(, 130.002) = 9.94, *p* = 0.002, *ƞ*_*p*_^*2*^ = 0.071, 95% CI [0.010, 0.169], and follow-up analyses clarified that the only significant trend driving the interaction was a positive relationship between chatbot exposure and perceived plausibility of AI origin for negative competence-related stimuli, *b* = 0.33, SE = 0.09, *p*_FDR_ = 0.002. No other trends were significant (Fig. 3B).

#### Attitude towards AI

The distributions of self-reported attitudes towards AI per study are shown in Fig. 3C. On average, participants in Studies 1, 2, and 3 held a positive opinion of AI in general (Study 1: *M* = 5.15, *SD* = 1.27; Study 2: *M* = 4.84, *SD* = 1.38; Study 3: *M* = 4.58, *SD* = 1.22), as suggested by one-sample one-sided t-tests against the scale midpoint (4) (Study 1: *t*(19) = 4.05,* p* < 0.001; Study 2: *t*(139) = 7.16,* p* < 0.001; Study 3: *t*(39) = 2.99,* p* = 0.002). A one-way ANOVA clarified that the average attitude towards AI did not differ between the three studies, *F*(2, 197) = 1.28, *p* = 0.279, *ƞ*_*p*_^*2*^ = 0.013. Additional analyses using the data from all studies revealed a small positive correlation between attitude towards AI and perceived plausibility that AI generated judgments in general, *r* = 0.16, 95% CI [0.023, 0.29], *t*(198) = 2.30,* p* = 0.023. Finally, we explored if the age of participants was negatively correlated with attitudes towards AI, but found no significant relationship between the variables, Pearson’s *r*(189) = 0.024, *p* = 0.737.

#### Beliefs about cues to AI

To gain insight into the heuristics that our participants might have relied upon during the task while guessing the source of the stimulus impressions, we collected both quantitative and qualitative data using a set of additional items.

*Quantitative.* All t-tests reported in these analyses are one-sample one-sided t-tests against the scale midpoint of four. In Studies 1 and 3, participants did not consider the length of an impression description to be an indication of AI origin, as mean ratings were both below the scale midpoint (Study 1: *M* = 3.80, *SD* = 1.88; Study 3: *M* = 3.80, *SD* = 1.76; both *ts* < 1). In Study 2, however, participants rated the length of an impression description as very indicative of AI origin (*M* = 4.46, *SD* = 1.80; *t*(140) = 3.04* p* = 0.001). In turn, across all three studies, participants considered that there were “patterns in the text” signaling AI origin, as suggested by ratings consistently and significantly higher than the scale midpoint (4) (Study 1: *M* = 5.20, *SD* = 1.61, *t*(19) = ,* p* = 0.002; Study 2: *M* = 5.78, *SD* = 1.18, *t*(140) = 17.94,* p* < 0.001; Study 3: *M* = 5.33, *SD* = 1.51, *t*(39) = 5.55,* p* < 0.001).

*Qualitative.* We used ChatGPT (v.3.5, Free Research Preview, August 3) as a text analysis tool to derive and summarize the main topics from participants’ responses regarding the diagnostic cues to the AI origin of impression descriptions. This analytical approach has recently been validated and shown to outperform other traditional and more laborious text analytical methods (Rathje et al., 2023). For more details about this analysis see Supplementary Information in the OSF repository. The resulting themes summarizing participants’ responses are listed in Table 3. In line with the quantitative measures, participants mentioned several cues related with form (e.g., length, structure, formality, word choices), some of which were likely made accessible by the items using response scales. Importantly, these data shed light on content-related cues. Of relevance to the present studies we highlight the emergence of themes like “personality and perspective” associating the absence of moral thinking or judgment to AI origin and a clear positioning in regard to some topic with human origin. The emergence of such a theme further clarifies the content of participants’ views about AI capabilities, particularly for morality-related judgments, as they hint at an inclination to find it less likely that AI generated morality-related content. Further corroborating this is the emergence of themes associating presence of emotional content in the target descriptions with a higher likelihood of human origin (i.e., “emotional content”); or associating human origin the expression of opinions with higher certainty (i.e., “opinions and assumptions”). These data, however, are less informative about any potential strategies used judge AI capabilities to assess competence.

**Table 3:** Summary of themes identified by ChatGPT regarding participants’ opinions on diagnostic cues to AI origin in impression descriptions (studies 2 and 3)

Theme	Summary
Language and writing style	Complex or technical vocabulary Formal and detached tone Use of unusual or "difficult" words Repetitive word patterns Use of phrases like "may be," "could be," "might be"
Emotional content	Lack of emotions or emotional understanding Absence of feelings or emotional aspect in descriptions Presence of emotions indicating human input
Sentence and text structure	Starting with an introduction and ending with a conclusion Overly detailed explanations Repetition of cues or phrases Excessive assumptions and drawn-out conclusions Repeating "Person X" frequently
Content focus	Detailed descriptions of various aspects of behavior Giving out conclusions or opinions about a person's behavior Moralizing and giving advice Lengthy texts with unnecessary information
Personality and perspective	AI tending to be neutral and non-judgmental Human descriptions containing more personal judgments Lack of moral thinking or judgment in AI-generated content
Word and term usage	Use of "Person X" instead of just "person" Defining or explaining terms within the description Use of generic and general explanations
Context and contextualization	Over-explaining situations or providing unnecessary context Lack of consideration for unnecessary aspects of behavior
Opinions and assumptions	AI tending to make assumptions and guesses Humans giving direct opinions or clear judgments
Formality and punctuation	Formal language and sentence construction Specific punctuation patterns
Personal connection	Human responses feeling more personal and relatable AI responses feeling like they were observing from the outside
Response length	AI tending to provide longer and more detailed responses

## Discussion

The present work aimed to investigate whether people perceive AI systems to be less capable of generating judgments about the morality of human behavior compared to generating written judgments about the competence of human behavior, when the true source of information is unknown. In light of the historical rise in public exposure to advanced AI technology occurring in parallel to the present work, we further explored the extent to which it impacted our participants’ perceptions of AI’s capabilities to assess human morality and competence. Across three studies, our results indicate that the degree to which a written impression about a particular human behavior was perceived to be of artificial origin depended on the social dimension (i.e., morality or competence) most strongly associated with the behavior under judgment. Specifically, participants tended to ascribe a lower plausibility of AI generation to descriptions of morality-related behaviors, compared to competence-related descriptions. However, this finding was unexpectedly and inconsistently dependent on the valence of stimuli, particularly when they were AI-generated in Studies 1 and 2. Thus, only partial support was obtained for our hypothesis. The current findings suggest that, when the true source of information is unknown, people will be more inclined to believe that competence-related judgments of human behaviors are AI-generated compared to morality-related judgments, especially when such judgments are about positive competence. This effect did not appear to depend on self-reported attitudes towards AI or confidence in one’s estimations about the artificial origin of judgments.

Importantly, our results provide initial insight into the content of stereotypical knowledge about AI at an important moment of history of AI development. This period is important in the sense that it captures one of the first massive infiltrations of AI into important domains of human activity (e.g., art, education), thereby opening ground for a higher degree of interaction between people and advanced AI. It was also during this period that state-of-the-art publicly available AI achieved the capability to convincingly mimic human moral judgments (Dillion et al., 2023). This raises the question of whether our results would capture any shift in people’s beliefs about AI capabilities to match humanlike behavior as they become increasingly exposed to them. The present findings suggest that people’s beliefs about AI capabilities for core social judgments remained largely invariant throughout the period under examination (between 1 and 6 months after launch of ChatGPT), and were only slightly impacted by previous (self-reported) interactions with chatbots including ChatGPT. Specifically, our exploratory analyses indicated that increased usage of chatbots like ChatGPT was associated with a higher tendency to attribute AI origin, but only significantly so for descriptions of negative competence. Although the remaining trends were not significant, the overall pattern of results appears to suggest that higher exposure to chatbots like ChatGPT is more clearly associated with an increased tendency to attribute AI origin to descriptions when they relate to competence, than when they relate to morality. For morality-related descriptions, there seems to be no impact of exposure, which may be suggesting that the tendency to attribute AI origin to morality or emotion laden assessments may be more resistant to change compared to competence or ability related assessments. Overall, however, an effect of dimension (or dimension and valence) was repeatedly detected throughout the timeline of our studies, despite of a massive increase in the public awareness and exposure to the capabilities of advanced AI (Ruby, 2023). The inherent uncertainty of our task along with the nature of our dependent measure (i.e., judgments of plausibility of category membership) are assumed to trigger reliance on the representativeness heuristic (Tversky & Kahneman, 1974), which in turn promotes reliance on stereotypical knowledge about the “human” and “AI” categories, over any other available relevant cues informing a more accurate judgment. In this context, the reliance on stereotypes about AI abilities may even be the only feasible approach to guessing the source of the impressions in an era where human-generated and AI-generated language became indistinguishable to humans (e.g., Jakesch et al., 2023). If our task is indeed capturing stereotypes about AI capabilities, likely formed through exposure to media and pop culture (e.g., Cross & Ramsey, 2021), we should expect them to be resistant to change and to change slowly over time (e.g., Eagly et al., 2020; Lai et al., 2016; Lippmann, 1922). The question remains whether these beliefs are more or less resistant to change compared to well-known rigid beliefs such as gender stereotypes (Fiske, 2017).

When explicitly asked about their beliefs about AI capabilities to judge morality and competence, participants attributed a higher capability to AI judgments of competence compared to AI judgments of morality. This suggests that, at least when directly asked about it, participants tended to attribute a lower ability to AI making moral judgments (vs. competence judgments) regardless of the valence associated with the behavior underlying the impression. Although this could be seen as reflecting an alignment between self-reported beliefs and the plausibility judgments made during the main task—where no information was explicitly given about the dimension or pole of stimuli—such an alignment only emerged between beliefs about AI capabilities for morality judgments and plausibility responses to morality-related stimuli. This could be suggesting that the plausibility judgments made during the main task were more strongly guided by beliefs in AI’s capability to assess morality than by beliefs in AI’s capability to assess competence. Such an interpretation fits well with the insights from the qualitative data showing how participants look for morality-related content to assess the likelihood of human origin. In fact, our qualitative data is in agreement with other studies examining the ability of LLMs to pass the Turing test (e.g., Jones & Bergen, 2023), by suggesting that the responses to the task were influenced not only by the association of the stimulus with morality or competence but also by less socially charged factors such as writing structure and style. In addition, the failure to find an alignment between the plausibility responses and the explicit beliefs in AI capabilities for judging competence could be related with some proclivity to focus on morality over competence information during the task. It may be that the ability to form moral judgments is seen as a more uniquely human ability like moral character or moral sensibility are perceived to be (e.g., Goodwin et al., 2014; Haslam, 2006). In turn, the failure to detect the same association for beliefs in AI capabilities to judge competence could be related to the tendency for people to focus primarily on information about morality (e.g., Wojciszke & Abele, 2008). This tendency could have promoted a strategy where participants focused on detecting human origin based on the presence of morality- or emotion-related cues in stimuli. Stimuli that did not incorporate these cues would then be rated as more likely to be AI-generated.

Overall, the pattern of results fits well with the documented human tendency to be averse to moral judgments generated by machines (Bigman & Gray, 2018), or the tendency for humans to prefer algorithmic advice in domains of high objectivity (e.g., financial advice) while preferring human advice in domains of high subjectivity like relationships (e.g., Castelo et al., 2019). The insights offered by the LLM-based analysis of participants’ beliefs about cues to AI origin, clarify that, besides aspects related to stylistic and structural features of the stimuli, any cue conveying an ability to experience, understand, and express emotion in judgments is considered by people to be an important cue to differentiate between AI and human origin. It seems conceivable that participants relied on the amount of emotional signal in the stimuli to inform their plausibility judgments, such that the less emotional signal, the less likely a stimulus was perceived to be AI-generated. Conversely, the more objective nature of an assessment of competence may have reduced the emotional content in competence-related stimuli, thus increasing their plausibility of AI origin. This could explain why morality-related stimuli tended to be perceived as less likely to be of AI origin, especially in light of prior work in mind perception and morality research documenting people’s tendency to ascribe a low ability to experience emotion to artificial entities (Bigman & Gray, 2018; Gray et al., 2007) and how such an ability is essential for moral reasoning (Gray et al., 2012; Haidt et al., 1993).

We did not expect people’s plausibility judgments to be more clearly differentiated between stimuli associated with positive morality and positive competence, compared to stimuli associated with negative morality and competence. This pattern suggests that people find it harder to assess the true origin (i.e., AI vs. human) of social judgments when these are about moral, immoral, or incompetent behavior, while finding it easier to attribute artificial origin to judgments of competent behavior. The current data only allow us to speculate on the reasons underlying this interaction pattern, or why it more clearly emerged for AI-generated materials. One possibility relates to potential differences between AI-generated and human-generated materials. One could argue that our AI-generated materials intentionally retain many cues to their origin, given the increasing pressure for AI developers to create transparent and explainable AI systems (e.g., Confalonieri et al., 2021). If the AI output is producing stronger cues about its origin for some types of behavior impressions than others, the observed results might then be simply reflecting that the true origin of some stimuli was easier to detect (e.g., judgments of high competence conveying more cues to AI than high morality stimuli). However, such an explanation does not align well with the emergence of a similar pattern of results with human-generated materials in Study 3 which, although non-significant, suggests some influence of the stimulus’ valence, even if subtle. The similarity of results across our studies using human-generated and AI-generated materials fits well with previous work showing that people rely on flawed strategies to differentiate between the human or the artificial origin of text (Jakesch et al., 2023), and with studies showing a tendency to attribute AI origin in tasks similar to ours, potentially as a strategy to avoid feeling deceived (see Jones & Bergen, 2023). Another potential factor driving the interaction could be any uncontrolled differences regarding the emotional signal conveyed by the different stimulus categories. Participants’ responses might have been more strongly driven by the emotional signal in a stimulus, than by the association of stimuli with specific social dimension (e.g., positive competence stimuli conveying less emotion than positive morality stimuli, but negative morality and negative competence stimuli conveying similar levels of emotion). Nevertheless, although the reasons behind the interaction remain unclear, our data offer some insight into the strategies that people use to assess the artificiality of judgments about human behavior, as well as the content of lay theories (i.e., stereotypical knowledge) about AI capabilities.

### Limitations and future directions

It is possible that the effects found in our studies could be driven by unknown specificities of the particular set of behaviors we used to generate both the AI-generated and human-generated impressions. Future studies should examine the generalizability of the present results with different sets of behaviors, ideally pretested on their morality and competence signal. These generated impressions could also be assessed on the same dimensions of their input behaviors, as well as on the emotional signal conveyed by the impression generator. Regarding the generation of descriptions, future studies should consider whether to rely on zero-shot or few-shot prompting techniques to generate the descriptions, ideally in a programmatically reproducible manner (e.g., using APIs). Another clear limitation of the present work relates to the relatively short time frame that encompasses the three studies. This brief time window might not allow us to capture any influence of the societal exposure to advanced AI on perceptions of AI capabilities to mimic unique human capabilities. With the fast pace of AI development observed in recent years, future similar studies might find it advantageous to rely exclusively on human-generated materials to overcome the lack of control over frequent upgrades to LLMs or their successors. In light of recent findings showing how perceptions of artificial agents as trustworthy does not predict intentions to rely on their judgments (Momen et al., 2023), follow-up studies should expand the focus to the behavioral implications of the current findings and explore the extent to which human rely on morality- or competence-related information of unknown origin to accomplish a given task. One such study could employ a trolley dilemma paradigm to assess how participants utilized morality and/ competence information about the potential victims under different conditions of certainty about the source of that information (i.e., Human, AI, both, or unknown origin). Another study could investigate the impact of manipulating the degree of uncertainty about the information generator (e.g., 50%-50%; 30% Human – 70% AI, 30% AI – 70% Human) on perceptions of AI origin, to investigate framing effects. Finally, cross-cultural studies would help to understand the extent to which the degree of acceptance and adoption of AI in different countries impacts the current findings.

## Conclusion

In sum, the current work adds to the emerging literature on perceptions of AI by offering initial insights into how humans estimate the capabilities of state-of-the-art AI to generate descriptions of judgments on important dimensions of human social evaluation (Abele et al., 2021; Fiske et al., 2007), and how these evolved in the first wave of mass public exposure to this technology. Although our results detected little to no impact in people’s perceptions of AI capabilities during the period we conducted our studies (first 6 months after ChatGPT was released), it should remain an open question whether more time and public exposure to advanced AI will impact people’s perceptions (or skepticism) about AI capabilities to mimic judgments on central dimensions of human social cognition.

## Data Availability

Data and materials can be found on this paper’s project page on the Open Science Framework: https://osf.io/r2t7b/

## Footnotes

1 These two dimensions have been differently labelled by different researchers (e.g., warmth and competence, morality and competence, agency and communion), but there is high overlap in their content across studies and time, and high consensus on what they represent (Abele et al., 2021). Further research has shown how morality and warmth/sociability represent two different facets of the broader communion/warmth dimension (Brambilla et al., 2011; Leach et al., 2007), and how competence and assertiveness reflect different facets of the broader agency/competence dimension (Abele et al., 2016; Carrier et al., 2014).

2 We understand this is not an optimal solution, and our decisions to collect the data under such circumstances were motivated by the risk of failing to collect the earliest possible perceptions of AI we could considering the rapid pace of development and regular improvement of ChatGPT and other Generative AI tools that we have been experiencing since the launch of ChatGPT.

3 Please note that in this context valence can be alternatively understood as the high and low poles of a dimension. The correspondence between the low and high poles with negative and positive, respectively, reflects the positive and linear relationship that characterizes the dimensions of morality and competence in the context of social evaluation (see e.g., Oliveira et al., 2020; Rosenberg et al., 1968; Suitner & Maass, 2008). For example, the negative behavior “Person X cheated in a card game while playing with a group of their friends” scores lowly on morality; and the positive behavior “Person X put money in the expired parking meter of a stranger” scores highly on morality. Although some readers familiarized with the social evaluation literature may prefer the use of dimension poles, so as to not confuse them with valence poles, here we opted to use valence given its direct correspondence with pole for these specific dimensions and to increase consistency of labels across the text.

## References

[CR1] Abele AE, Cuddy AJC, Judd CM, Yzerbyt VY (2008). Fundamental dimensions of social judgment. European Journal of Social Psychology.

[CR2] Abele AE, Ellemers N, Fiske ST, Koch A, Yzerbyt V (2021). Navigating the social world: Toward an integrated framework for evaluating self, individuals, and groups. Psychological Review.

[CR3] Abele AE, Hauke N, Peters K, Louvet E, Szymkow A, Duan Y (2016). Facets of the fundamental content dimensions: Agency with competence and assertiveness—Communion with warmth and morality. Frontiers in Psychology.

[CR4] Argyle LP, Busby EC, Fulda N, Gubler JR, Rytting C, Wingate D (2023). Out of one, many: Using language models to simulate human samples. Political Analysis.

[CR5] Barr DJ, Lev R, Scheepers C, Tily HJ (2013). Keep it maximal appendix. Journal of Memory and Language.

[CR6] Bates D, Mächler M, Bolker B, Walker S (2015). Fitting Linear Mixed-Effects Models using lme4. Journal of Statistical Software.

[CR7] Benjamini Y, Hochberg Y (1995). Controlling the false discovery rate: A practical and powerful approach to multiple testing. Journal of the Royal Statistical Society.

[CR8] Bigman YE, Gray K (2018). People are averse to machines making moral decisions. Cognition.

[CR9] Binz M, Schulz E (2023). Using cognitive psychology to understand GPT-3. Proceedings of the National Academy of Sciences.

[CR10] Borkenau P (1986). Toward an understanding of trait interrelations: Acts as instances for several traits. Journal of Personality and Social Psychology.

[CR11] Brambilla M, Rusconi P, Sacchi S, Cherubini P (2011). Looking for honesty: The primary role of morality (vs. Sociability and competence) in information gathering. European Journal of Social Psychology.

[CR12] Cameron CD, Lindquist KA, Gray K (2015). A constructionist review of morality and emotions: No evidence for specific links between moral content and discrete emotions. Personality and Social Psychology Review.

[CR13] Carrier A, Louvet E, Chauvin B, Rohmer O (2014). The primacy of agency over competence in status perception. Social Psychology.

[CR14] Castelo N, Bos MW, Lehmann DR (2019). Task-dependent algorithm aversion. Journal of Marketing Research.

[CR15] Confalonieri R, Coba L, Wagner B, Besold TR (2021). A historical perspective of explainable Artificial Intelligence. Wires Data Mining and Knowledge Discovery.

[CR16] Cross ES, Ramsey R (2021). Mind meets machine: Towards a cognitive science of human–machine interactions. Trends in Cognitive Sciences.

[CR17] Darda K, Carre M, Cross E (2023). Value attributed to text-based archives generated by artificial intelligence. Royal Society Open Science.

[CR18] DeBruine LM, Barr DJ (2021). Understanding mixed-effects models through data simulation. Advances in Methods and Practices in Psychological Science.

[CR19] Dijkstra JJ (1999). User agreement with incorrect expert system advice. Behaviour & Information Technology.

[CR20] Dillion D, Tandon N, Gu Y, Gray K (2023). Can AI language models replace human participants?. Trends in Cognitive Sciences.

[CR21] Eagly AH, Nater C, Miller DI, Kaufmann M, Sczesny S (2020). Gender stereotypes have changed: A cross-temporal meta-analysis of U.S. public opinion polls from 1946 to 2018. American Psychologist.

[CR22] Fazio RH (2007). Attitudes as object–evaluation associations of varying strength. Social Cognition.

[CR23] Fiske ST (2017). Prejudices in cultural contexts: Shared stereotypes (gender, age) versus variable stereotypes (race, ethnicity, religion). Perspectives on Psychological Science.

[CR24] Fiske ST (2018). Stereotype content: Warmth and competence endure. Current Directions in Psychological Science.

[CR25] Fiske ST, Cuddy AJC, Glick P (2007). Universal dimensions of social cognition: Warmth and competence. Trends in Cognitive Sciences.

[CR26] Freeman JB, Ambady N (2011). A dynamic interactive theory of person construal. Psychological Review.

[CR27] Fumagalli M, Ferrucci R, Mameli F, Marceglia S, Mrakic-Sposta S, Zago S, Lucchiari C, Consonni D, Nordio F, Pravettoni G, Cappa S, Priori A (2010). Gender-related differences in moral judgments. Cognitive Processing.

[CR28] Gangadharbatla H (2022). The role of AI attribution knowledge in the evaluation of artwork. Empirical Studies of the Arts.

[CR29] Gilbert DT, Gilbert DT, Fiske ST, Lindzey G (1998). Ordinary personology. The handbook of social psychology.

[CR30] Goodwin GP, Piazza J, Rozin P (2014). Moral character predominates in person perception and evaluation. Journal of Personality and Social Psychology.

[CR31] Gray HM, Gray K, Wegner DM (2007). Dimensions of mind perception. Science.

[CR32] Gray K, Young L, Waytz A (2012). Mind perception is the essence of morality. Psychological Inquiry.

[CR33] Greene JD, Sommerville RB, Nystrom LE, Darley JM, Cohen JD (2001). An fMRI investigation of emotional engagement in moral judgment. Science.

[CR34] Gunser VE, Gottschling S, Brucker B, Richter S, Gerjets P, Stephanidis C, Antona M, Ntoa S (2021). Can users distinguish narrative texts written by an artificial intelligence writing tool from purely human text?. HCI international 2021—posters.

[CR35] Haidt J, Koller SH, Dias MG (1993). Affect, culture, and morality, or is it wrong to eat your dog?. Journal of Personality and Social Psychology.

[CR36] Haslam N (2006). Dehumanization: An integrative review. Personality and Social Psychology Review.

[CR37] Hitsuwari J, Ueda Y, Yun W, Nomura M (2023). Does human–AI collaboration lead to more creative art? Aesthetic evaluation of human-made and AI-generated haiku poetry. Computers in Human Behavior.

[CR38] Hortensius R, Cross ES (2018). From automata to animate beings: The scope and limits of attributing socialness to artificial agents: Socialness attribution and artificial agents. Annals of the New York Academy of Sciences.

[CR39] Horton, J. J. (2023). *Large language models as simulated economic agents: What can we learn from homo silicus?*10.48550/ARXIV.2301.07543

[CR40] Hu, K. (2023, February 2). *ChatGPT sets record for fastest-growing user base—Analyst note*. Reuters. https://www.reuters.com/technology/chatgpt-sets-record-fastest-growing-user-base-analyst-note-2023-02-01/

[CR41] Jakesch M, Hancock JT, Naaman M (2023). Human heuristics for AI-generated language are flawed. Proceedings of the National Academy of Sciences.

[CR42] Jones, C., & Bergen, B. (2023). *Does GPT-4 pass the Turing test?*10.48550/ARXIV.2310.20216

[CR43] Judd CM, Garcia-Marques T, Yzerbyt VY (2019). The complexity of relations between dimensions of social perception: Decomposing bivariate associations with crossed random factors. Journal of Experimental Social Psychology.

[CR44] Judd CM, James-Hawkins L, Yzerbyt V, Kashima Y (2005). Fundamental dimensions of social judgment: Understanding the relations between judgments of competence and warmth. Journal of Personality and Social Psychology.

[CR45] Kervyn N, Bergsieker HB, Fiske ST (2012). The innuendo effect: Hearing the positive but inferring the negative. Journal of Experimental Social Psychology.

[CR46] Kervyn N, Fiske ST, Yzerbyt VY (2013). Integrating the stereotype content model (warmth and competence) and the Osgood semantic differential (evaluation, potency, and activity). European Journal of Social Psychology.

[CR47] Köbis N, Mossink LD (2021). Artificial intelligence versus Maya Angelou: Experimental evidence that people cannot differentiate AI-generated from human-written poetry. Computers in Human Behavior.

[CR48] Kuznetsova A, Brockhoff PB, Christensen RHB (2017). lmerTest package: Tests in linear mixed effects models. Journal of Statistical Software.

[CR49] Lai, C. K., Skinner, A. L., Cooley, E., Murrar, S., Brauer, M., Devos, T., Calanchini, J., Xiao, Y. J., Pedram, C., Marshburn, C. K., Simon, S., Blanchar, J. C., Joy-Gaba, J. A., Conway, J., Redford, L., Klein, R. A., Roussos, G., Schellhaas, F. M. H., Burns, M., … Nosek, B. A. (2016). Reducing implicit racial preferences II: Intervention effectiveness across time. *Journal of Experimental Psychology. General*, *145*(8), 1001–1016. 10.1037/xge0000179 10.1037/xge0000179 27454041

[CR50] Leach C, Ellemers N, Barreto M (2007). Group virtue: The importance of morality (vs. Competence and sociability) in the positive evaluation of in-groups. Journal of Personality and Social Psychology.

[CR51] Lee MK (2018). Understanding perception of algorithmic decisions: Fairness, trust, and emotion in response to algorithmic management. Big Data & Society.

[CR52] Lenth, R. V. (2022). *emmeans: Estimated Marginal Means, aka Least-Squares Means* (R package version 1.8.3) [Computer software].

[CR53] Lippmann W (1922). Public opinion.

[CR54] Luke SG (2017). Evaluating significance in linear mixed-effects models in R. Behavior Research Methods.

[CR55] Mickelberg A, Walker B, Ecker UKH, Howe P, Perfors A, Fay N (2022). Impression formation stimuli: A corpus of behavior statements rated on morality, competence, informativeness, and believability. PLoS ONE.

[CR56] Momen, A., De Visser, E., Wolsten, K., Cooley, K., Wallisser, J., & Tossell, C. C. (2023). *Trusting the moral judgments of a robot: Perceived moral competence and humanlikeness of a GPT-3 enabled AI*. 501–510

[CR57] Nightingale SJ, Farid H (2022). AI-synthesized faces are indistinguishable from real faces and more trustworthy. Proceedings of the National Academy of Sciences of the United States of America.

[CR58] Nisbett RE, Wilson TD (1977). The halo effect: Evidence for unconscious alteration of judgments. Journal of Personality and Social Psychology.

[CR59] Oliveira M, Garcia-Marques T, Garcia-Marques L, Dotsch R (2020). Good to Bad or Bad to Bad? What is the relationship between valence and the trait content of the Big Two?. European Journal of Social Psychology.

[CR60] OpenAI. (2022). *ChatGPT* (December 15) [Large language model; Large language model]. https://chat.openai.com/chat

[CR61] Osgood CE, Suci GJ, Tannenbaum PH (1957). The measurement of meaning.

[CR62] R Core Team. (2022). *R: A language and environment for statistical computing* [Computer software]. R Foundation for Statistical Computing.

[CR63] Rathje, S., Mirea, D.-M., Sucholutsky, I., Marjieh, R., Robertson, C., & Van Bavel, J. J. (2023). *GPT is an effective tool for multilingual psychological text analysis* [Preprint]. PsyArXiv. 10.31234/osf.io/sekf5 10.1073/pnas.2308950121 PMC11348013 39133853

[CR64] Rosenberg S, Nelson C, Vivekananthan PS (1968). A multidimensional approach to the structure of personality impressions. Journal of Personality and Social Psychology.

[CR65] Rosenberg S, Olshan K (1970). Evaluative and descriptive aspects in personality perception. Journal of Personality and Social Psychology.

[CR66] Ruby, D. (2023, May 18). 57+ ChatGPT statistics 2023. *DemandSage*. https://www.demandsage.com/chatgpt-statistics/

[CR67] Shank DB, Graves C, Gott A, Gamez P, Rodriguez S (2019). Feeling our way to machine minds: People’s emotions when perceiving mind in artificial intelligence. Computers in Human Behavior.

[CR68] Stolier RM, Hehman E, Keller MD, Walker M, Freeman JB (2018). The conceptual structure of face impressions. Proceedings of the National Academy of Sciences.

[CR69] Suitner C, Maass A (2008). The role of valence in the perception of agency and communion. European Journal of Social Psychology.

[CR70] Tiku, N. (2022, June 11). *The Google engineer who thinks the company’s AI has come to life* [News]. The Washington Post. https://www.washingtonpost.com/technology/2022/06/11/google-ai-lamda-blake-lemoine/

[CR71] Tucciarelli R, Vehar N, Chandaria S, Tsakiris M (2022). On the realness of people who do not exist: The social processing of artificial faces. iScience.

[CR72] Tversky A, Kahneman D (1974). Judgment under uncertainty: Heuristics and biases. Science.

[CR73] Wegner DM, Gray K (2017). The mind club: Who thinks, what feels, and why it matters.

[CR74] Westfall J, Kenny DA, Judd CM (2014). Statistical power and optimal design in experiments in which samples of participants respond to samples of stimuli. Journal of Experimental Psychology: General.

[CR75] Wojciszke B (2005). Morality and competence in person- and self-perception. European Review of Social Psychology.

[CR76] Wojciszke B, Abele AE (2008). The primacy of communion over agency and its reversals in evaluations. European Journal of Social Psychology.

[CR77] Yzerbyt VY, Kervyn N, Judd CM (2008). Compensation versus halo: The unique relations between the fundamental dimensions of social judgment. Personality and Social Psychology Bulletin.

